# A Case of Poisoning with Morphia and Hyd. Chloral—Remarkable Recovery

**Published:** 1872-06-15

**Authors:** Theodore Griffin

**Affiliations:** St. Louis, Mo.


### A CASE OF POISONING WITH MORPHIA AND HYD. CHLORAL—REMARKABLE RECOVERY.

BY THEODORE GRIFFIN, M.D., OF ST. LOUIS, MO.

In the July number of the Chicago MEDICAL EXAMINER for 1871, I reported a case of opium poisoning successfully treated by the hypodermic injection of *atropine*. That case was regarded as one of unusual interest, from the fact that the patient was very nearly dead when the remedy was used, there being only about two imperfect respirations per minute, with every other evidence of rapidly approaching death. In this case no other remedy than the atropine hypodermically used was applied.

I wish here to report a case of recovery from poisoning with morphia and hyd. chloral, which, so far as my knowledge extends, is the most remarkable on record, and which forcibly illustrates the necessity of persisting in the vigorous employment of the means of resuscitation so long as there are perceptible contractions of the heart.

On the 2d of May, 1872, I was called to see Mrs. G. of St. Louis, aged about 30 years, of medium hight, and rather obese. I found her alone in her room, and in a state of partial unconsciousness, with a quiet muttering delirium. I supposed her to be under the influence of liquor, and information which I afterwards received confirmed me in this belief. Domestic trouble had doubtless led her to unusual excesses; her husband having deserted her two weeks previous to this time. She was suffering from much nervous and general physical debility, delirium and sleeplessness. Accordingly, tinct. of nux vomica as a nervous tonic and stimulant was prescribed; and for the insomnia hyd. chloral ʒ 1, tinct. opii ʒ 1, mix, which she took in divided doses on the night of the 2d, without its producing any sedative effect whatever. On the morning of the 3d her general symptoms were much improved, and she was told that on the succeeding night she would probably sleep without an anodyne being administered. Yet, on the morning of the 4th she sent to my office the statement that she had not slept any during the past night, and requested that I should prescribe morphine for her. Regarding the case as one of rather obstinate insomnia, resulting from *delirium ebriosum*, I prescribed as follows:

℞ Sulph. morph. gr. viij.

Hyd. chloral ʒ 1.

Aqua camph. ℥ 1.

Syr. simplex ℥ 1. Mix.

Of this mixture she was directed to take one teaspoonful every hour until sleeping. She took the first dose at 10 a.m. on the 4th. At 12 o'clock I was called in haste to see her. I found her profoundly under the influence of the narcotic medicines, and upon examining the bottle which contained the medicine, it was found that she had taken nearly three-fourths of its contents during the two hours, making about six grains of morphia and forty grs. hyd. chloral. The ordinary treatment in these cases was at once vigorously resorted to. At 2 o'clock she was profoundly comatose. Drs. W. Johnston and Grissom were called to my aid. To my suggestion that atropine be used hypodermically Dr. Johnson acceded. I immediately procured the following solution: Atropia gr. 1, aqua ℥ 1. Of this solution I presume one-half was injected near the median basilic vein by Dr. Grissom. Some of the solution was wasted, as the syringe worked imperfectly, so that it is impossible to state the exact quantity used. The injection of atropia was given at about 2 o'clock; a few moments before the use of the atropia artificial respiration was begun by alternately elevating the arms and depressing the chest.

The patient presented the following symptoms at 4 o'clock p.m.: Lying on her back on the floor, lips and face of a dark purple color; extreme capillary congestion of the surface; eyes fixed; pulse barely perceptible, beats 140 times per minute, and very feeble; no respirations appreciable; tongue falls back into the pharynx. The tongue was drawn forward and artificial respiration vigorously continued. 4 o'clock p.m.—The pupils are now widely dilated by the atropia; pulse scarcely perceptible, at times absent; sordes accumulating on the teeth; no respirations appreciable; artificial respiration continued. 5 o'clock p.m.—Pulse more easily detected; no respirations appreciable; artificial respirations continued. At about this time ten or twelve medical students arrived and assisted. 6 o'clock p. m. — Condition unchanged. 7 o'clock p.m.—Decided improvement in her circulation; her symptoms in other respects are apparently unchanged; artificial respiration continued. 8 o'clock p.m.—Pupils not so much dilated; pulse 130 and more full; color of skin and lips much improved; no respirations are yet appreciable, yet hopes are now entertained of her recovery; artificial respiration continued. 8½ o'clock p.m.—Now, for the first time since 2½ o'clock, a period of six hours, it is evident that she breathes; the respirations are short, jerking, and otherwise imperfect, yet there are sixteen of them in a minute; pulse 130 and distinct; pupils well dilated; artificial respiration discontinued. From this time she gradually improved. At the suggestion of Dr. Grissom injections per rectum of strong warm coffee were given; one and a half quarts were administered in this way during an interval of four hours, with some apparent benefit.

At 3½ o'clock a.m. of the 5th, she was taken from the floor and placed upon a bed; pupils still dilated. She has at short intervals periods of wandering delirium, with periods of consciousness. At 4 o'clock a.m. I left the patient in charge of four students. I visited her again at 7 a.m. and found her quite intelligent, respirations natural, pulse 100 and full, pupils somewhat dilated. Her skin is hot, and she complains much of muscular soreness, of pain in the chest, and coughs some. Of her subsequent treatment it would be uninteresting to speak. She necessarily suffered considerably from injuries received during the continuous employment of artificial respiration for a period of six hours. Nourishment and rest were the principal remedies indicated. On the morning of the 6th I had occasion to visit the country and requested Dr. Grissom to attend the patient during my absence. When I returned, after an absence of three days, I found that my services were not further desired, the case passed into the hands of Dr. Grissom.

This case doubtless illustrates, in an unparalleled degree, the efficiency of prolonged and unremitting artificial respiration in cases of poisoning by narcotic medicines, even when all signs of vitality are extinct (except, perhaps, contractions of the heart).

Death from opium poisoning begins at the brain; the heart is the last vital organ to cease its functions; it derives most of its supply of nervous influence from the sympathetic nervous system, whose plexuses (cardiac) lie in close proximity to the heart, and whose filaments permeate almost every square line of its substance. By some, the various ganglia of the organic nervous system are supposed to be centers of independent nervous force; little brains, as it were, and that they can give and receive impressions, and sustain vitality in a measure independent of the cerebro-spinal system. If this theory be true, it accounts in a measure for the fact that when the brain and its nervous appendages, with all their dependent organs, are overwhelmed and stilled by the poisonous influence of opium, the heart continues to beat and the circulation to go on—presenting the phenomenon of a living heart in a dead body. Artificial respiration, by bringing air in contact with the pulmonary circulation, liberates the carbonic acid with which the blood quickly becomes loaded and supplies to it oxygen; which when returned to the heart, stimulates it to more vigorous contraction, supplying thus oxygenated blood to a poisoned brain. Thus the blood is oxygenized, thus the heart is stimulated and its action kept up; thus the brain and nervous centers are supplied with arterial blood, until the narcotic has expended its power and life is restored.

How far the atropine acted as an antidote in this case cannot of course be known; all we know is that its physiological effect as manifested by dilated pupils, was apparent in fifteen minutes after its injection, and continued until after her recovery from the narcotism.

Doubtless I committed an error in placing so large an amount of morphine in such hands. The dose prescribed may be regarded as unsafe; yet every medical man will admit that no one is so competent to judge of the amount of morphine required in a given case as the attending physician, circumstances so alter cases. Dr. Grissom procured the invaluable assistance of the students, and by united and constant muscular labor for a period of six hours and a half a life was saved.

